# Role of Chest Computed Tomography versus Real Time Reverse Transcription Polymerase Chain Reaction for Diagnosis of COVID-19: A Systematic Review and Meta-Analysis

**DOI:** 10.1155/2021/8798575

**Published:** 2021-05-28

**Authors:** Dina M. Ali, Lamiaa G. Zake, Nevine K. El Kady

**Affiliations:** ^1^Tropical Medicine Department, Faculty of Human Medicine, Zagazig University, Zagazig, Egypt; ^2^Chest Department, Faculty of Human Medicine, Zagazig University, Zagazig, Egypt; ^3^Forensic and Clinical Toxicology Department, Faculty of Human Medicine, Cairo University, Cairo, Egypt

## Abstract

**Background:**

The current global pandemic of COVID-19 is considered a public health emergency. The diagnosis of COVID-19 depends on detection of the viral nucleic acid by real time reverse transcription polymerase chain reaction (RT-PCR). However, false-negative RT-PCR tests are reported and could hinder the control of the pandemic. Chest computed tomography could achieve a more reliable diagnosis and represent a complementary diagnostic tool.

**Aim:**

To perform a meta-analysis and systematic review to find out the role of chest computed tomography versus RT-PCR for precise diagnosis of COVID-19 infection.

**Methods:**

We searched three electronic databases (PubMed, ScienceDirect, and Scopus) from April 1 to April 20, 2020, to find out articles including the accuracy of chest computed tomography scan versus RT-PCR for diagnosis of SARS-CoV-2 infection. Observational studies, case series, and case reports were included.

**Results:**

A total of 238 articles were retrieved from the search strategy. Following screening, 39 articles were chosen for full text assessment and finally 35 articles were included for qualitative and quantitative analysis. Chest computed tomography showed a wide range of sensitivity varied from 12%–100%.

**Conclusion:**

Chest computed tomography is playing a key role for diagnosis and detection of COVID-19 infection. Computed tomography image findings may precede the initially positive RT-PCR assay.

## 1. Introduction

In December 2019, a group of patients with pneumonia of unknown etiology had been reported in Wuhan of China [1]. A new coronavirus was identified as the causative pathogen. The Coronavirus Study Group (CSG) of the international committee on taxonomy of the viruses recognized this virus as a close variant of Severe Acute Respiratory Syndrome Coronavirus (SARS-CoV) and named it as SARS-CoV-2 [2]. The World Health Organization (WHO), on January 30, 2020, announced the new SARS-CoV-2 outbreak as a Public Health Emergency of International Concern (PHEIC) [3].

It is very essential to diagnose precisely the suspected patients with COVID-19 infection for opportune isolation and treatment. The current recommendations are to use the RT-PCR for identification of SARS-CoV-2 in the respiratory specimens. However, there are already more than seven different SARS-CoV-2 RT-PCR tests [4] and the currently available kits provide variable sensitivities ranging between 45% and 60%. Thus, repeated RT-PCR testing may be required to establish the diagnosis particularly early in the course of infection [5]. Data have emerged on the usefulness of chest CT scan for early diagnosis of cases. Several studies published to date have revealed higher sensitivity of chest CT for detection of SARS-CoV-2 infection in comparison to the RT-PCR testing [6].

We aimed to carry out a systematic review and meta-analysis to find out the role of chest CT scan versus RT-PCR for precise diagnosis of COVID-19 infection.

## 2. Methods

### 2.1. Search Strategy

The present analysis was performed according to the Preferred Reporting Items for Systematic Reviews and Meta-Analysis (PRISMA) guidelines. Electronic search, including three databases (PubMed, ScienceDirect, and Scopus), was conducted during April, 2020. The following MeSH keywords and terms were used, “COVID-19,” “Chest CT,” and “RT-PCR.” Following the retrieval of relevant articles, reference lists from sourced publications were checked to confirm that all available evidence is covered.

### 2.2. Study Selection

**Figure 1:**
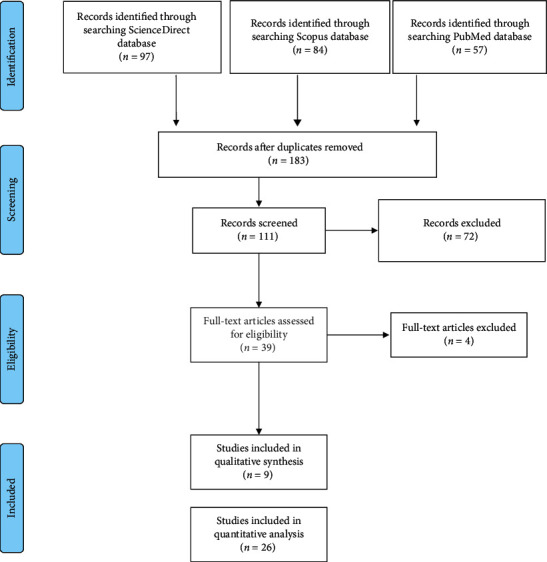
PRISMA flow chart for systematic review of studies identifying chest CT versus RT-PCR.

The outcome of the primary search was first screened by the title and abstract. Then, the authors made certain the included studies are similar enough to be easily comparable [7]. After retrieval, duplicates were withdrawn, and all publications were fully reviewed to ensure they met the eligibility criteria. Assessment of full text articles was done by two independent reviewers, and any disagreements were resolved by discussion. The identification and screening process is presented in Figure 1.

### 2.3. Inclusion and Exclusion Criteria

Authors decided to include articles if they fulfilled the following criteria:

  Published between December 2019 and 2020.
  Available in English language.

Studies were excluded if the following fulfilled:

  Did not directly engage with the topic.
  Not in English language.
  Studies included pregnant females.
  Studies included children and neonates.

### 2.4. Data Extraction Process

Data extraction form including the country, the date and year of publication, the number of cases, age, sex, and outcome (e.g., sensitivity and specificity of chest CT) were fulfilled. Data extraction and analysis were centered on the findings and the discussion sections of the included articles.

### 2.5. Assessment of Methodological Quality

The Institute of Health Economics (IHE) quality appraisal checklist for case series studies was used to assess the methodological strengths and weaknesses of the included studies [8]. The authors evaluated each study against these criteria.

## 3. Results

**Figure 2:**
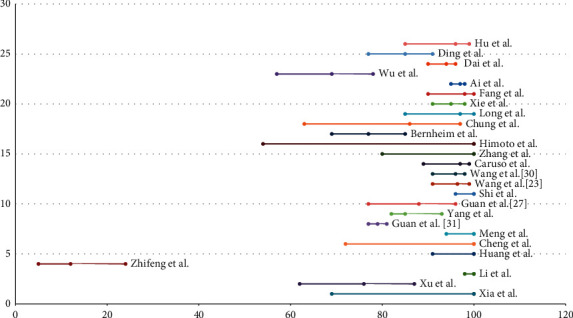
Forest plot showing chest CT sensitivity (95% CI) for all included studies.

A total of 238 articles were retrieved from the search strategy including 2 case series and 7 case reports. After title and abstract screening, 39 articles were chosen for full text assignment. Of these, 4 articles were excluded. Nine articles were included for qualitative analysis and 26 articles for quantitative analysis. The main characteristics of the included articles, case reports, and case series are summarized in Tables 1 and 2. Most articles are from China (24 studies), one study from Italy, and one study from Japan. The total patients included are 4250 patients, of them 2122 were males (49.9%) and 2128 (50.1%) females. Most of the studies are retrospective cohort. The case reports included 5 reports from China, one report from Korea, and one from Indonesia. The total number of patients included in case reports are 11 patients, 6 males (54.5%) and 5 females (45.5%). Two case series are available, one from USA and one from France. We analyzed one variable for the meta-analysis which is the sensitivity of chest CT compared to RT-PCR as the gold standard test as shown in Table 3 and Figure 2.

## 4. Discussion

Based on the latest guidelines for diagnosis and treatment of the novel coronavirus pneumonia released by Chinese authorities, the diagnosis of COVID-19 infection must be confirmed by RT-PCR which is considered the gold standard test [9]. However, its relatively long processing time can interfere with the control of the disease epidemic. Additionally, several elements may influence RT-PCR test results such as specimen type (upper or lower respiratory tract) and collection procedures, as well as the performance of the detection kits [10].

Chest CT scan is a routine, fast, and easy to perform imaging tool for diagnosis of pneumonia. It may provide benefit for diagnosis of COVID-19 taking into consideration that almost all COVID-19 patients demonstrate typical radiographic features including GGOS, peripheral multifocal patchy consolidation, and/or interstitial changes [11].

### 4.1. Analysis of Research Articles

#### 4.1.1. Consistency between Chest CT and RT-PCR

Cheng et al. studied chest CT manifestations for eleven laboratory-confirmed COVID-19 patients and all of them (11/11) demonstrated chest CT finding abnormalities [12]. Huang et al. as well declared that the entire 41 laboratory-confirmed cases for COVID-19 infection had pneumonia with abnormal findings on chest CT [13]. Along the same line, Shi et al. reported 81 patients with confirmed PT-PCR COVID-19 infection and all the included patients had abnormal chest CT findings [14].

Clinical and imaging features for 10 patients with COVID-19 laboratory-confirmed infection were reported by Xia et al. [15] and all studied patients had abnormal chest CT findings. Additionally, Li et al. assessed the clinical characteristics of 225 laboratory-confirmed cases in a tertiary hospital near Wuhan, China, and chest CT for all studied cases demonstrated lung infiltrates [16].

Furthermore, Meng et al. characterized the chest CT findings for 58 asymptomatic patients with laboratory-confirmed infection. All the included patients showed chest CT finding abnormalities. Thus, we must pay attention to asymptomatic infections which act as covert transmitter of infection. Taking into consideration that some asymptomatic patients can deteriorate in short duration, the surveillance of asymptomatic patients with COVID-19 is therefore crucial [17].

Himoto et al. evaluated the diagnostic performance of chest CT for diagnosing COVID-19 infection in Japan. The study included 21 patients with clinically suspected infection. Only 6/21 (28.5%) patients showed positive RT-PCR and were confirmed for COVID-19 infection. The entire six laboratory-confirmed cases showed chest CT images consistent with COVID-19 infection. Thus, according to Himoto et al., chest CT reported a sensitivity of 100% for diagnosis of COVID-19 infection and therefore could play an important supplemental role to triage and detect patients with suspected COVID-19 pneumonia before getting the results of RT-PCR [18].

Moreover, Zhang et al. studied the radiological features of high-resolution chest CT done for 17 laboratory-confirmed patients for COVID-19 infection by RT-PCR. All patients expressed radiological changes on initial CT examination (sensitivity 100%) and the authors recommended that chest CT examination may aid in the rapid identification of likely infection and guide patient management decisions [19].

#### 4.1.2. Discrepancy between Chest CT and RT-PCR

Ai et al. studied the correlation of chest CT and RT-PCR testing. Their study included 1014 suspected COVID-19 cases. Twenty-one (21/1014, 2%) patients had positive RT-PCR results but without abnormalities on initial CT examination. The chest CT features of (308/1014, 30%) patients suggested COVID-19, while their RT-PCR assays from oropharyngeal specimens were negative. The authors stated that positive chest CT findings despite of negative RT-PCR test results can still be highly indicative of COVID-19 infection [6].

Fang et al. conducted a study on 51 patients, 36/51 (70.5%) had initial positive RT-PCR, while 12/51 patients were confirmed by two RT-PCR tests, 2/51 patients by three tests, and 1 patient by four tests. Therefore, according to Fang et al., a single respiratory swab offers a positivity rate of 70%, an extra 24% after a second test, and an additional 3.9% after a third one. On the contrary, 98% of the patients had abnormal chest CT scan findings compatible with viral pneumonia [20].

Xie et al. conducted another study involving 167 laboratory-confirmed cases. For 155/167 patients (93%), both RT-PCR and chest CT were concordant for COVID-19 infection. In 7 patients (4%), RT-PCR was positive with initially negative chest CT scan. Five patients (3%) were with negative initial RT-PCR and positive CT scan [21].

Long et al.'s study included 36 cases with laboratory-confirmed COVID-19 infection. Twenty-nine (29/36, 80.5%) patients had positive chest CT findings and initially positive RT-PCR. Six (6/36, 16.7%) patients were with negative RT-PCR and positive CT findings at initial presentation, only one (1/36, 2.7%) patient was observed with positive RT-PCR but negative chest CT findings. The authors reported that considering false-negative results of RT-PCR and its relatively long processing time, the patients with typical chest CT findings should be isolated and repeated RT-PCR is required to avoid misdiagnosis [22].

Wang et al. reported 110 (96.5%) patients with abnormal chest CT findings out of 114 patients with confirmed COVID-19 infection. According to Wang et al. spiral chest CT is a sensitive examination method and can be applied to make an early diagnosis, evaluation of disease progression, with a diagnostic sensitivity and accuracy better than that of nucleic acid detection [23].

Yang et al. analyzed the radiological characteristics of 149 laboratory-confirmed cases. Only 17/149 (11.4%) had initially negative chest CT findings. Among these 17 patients, the chest CT of 12 patients kept being negative, with the latest follow-up CT 10.3 days later. In contrast, the chest CT of the other 5 patients became positive after about 7 days [24].

Caruso et al.'s study is the only clinical study from Italy available in the literature. The authors compared the accuracy of chest CT with RT-PCR. Using RT-PCR as a reference standard, sensitivity, specificity, and accuracy of chest CT were 97%, 56%, and 72%, respectively. Thus, chest CT demonstrated high sensitivity (97%) but lower specificity (56%) [25]. On the other hand, Xu et al. studied the clinical features of 51 laboratory-confirmed SARS-CoV-2 infection from China. They reported an overall chest CT sensitivity of 76.4% when RT-PCR was used as the gold standard test [26].

Guan et al. evaluated the radiological features of 53 confirmed cases by RT-PCR. Among the 53 cases, pneumonia was absent in the initial chest CT examination of 6 patients but 2 of the 6 patients showed pneumonia during follow-up. The researchers recommended that chest CT scan can quickly identify suspected patients and help significantly to isolate the source of infection, cutting off the route of transmission and avoiding further spread of infection [27].

Chung et al. studied the chest CT imaging features of 21 laboratory-confirmed COVID-19 infection by RT-PCR. Eighteen (18/21, 85.7%) patients showed positive CT findings with initial positive RT-PCR. Three cases (3/21, 14.3%) showed positive RT-PCR but negative chest CT findings. One of the three patients progressed and developed a solitary rounded GGO lesion but the others underwent follow-up chest CT four days after the initial CT examination that remained entirely normal. Thus, a sensitivity rate of 85.7% for chest CT was reported in this study [11].

Bernheim et al. evaluated the chest CT findings in 121 patients with confirmed laboratory COVID-19 infection by RT-PCR testing. They reported 27/121 patients (22%) with normal chest CT images and positive RT-PCR. Thus, the recorded sensitivity for chest CT according to Bernheim et al. is 77.6% [28].

Wu et al. conducted a descriptive study for clinical, laboratory, and imaging features for confirmed cases of COVID-19 infection. Their study included 80 laboratory-confirmed cases by RT-PCR. Fifty-five (55/80, 68.7%) patients had abnormal chest CT imaging and twenty-five (25/80, 31%) patients had initially normal chest CT with positive RT-PCR. Hence, the authors suggested that chest imaging should be combined for comprehensive analysis [29].

Wang et al. reported that only 5/125 (4%) laboratory-confirmed cases showed no abnormal findings on chest CT scan [30]. Additionally, Guan et al. studied the clinical characteristics of 1099 COVID-19 confirmed cases by RT-PCR in China and reported that 230/1099 had normal radiological findings by both chest radiograph and chest CT [31].

On the contrary, Zhifeng et al. reported the lowest sensitivity for chest CT in comparison to the gold standard RT-PCR. The sensitivity of chest CT was 12% (95% CI: 4.6–24.3) while the sensitivity of RT-PCR testing was 30.16% (95% CI: 19.2–43) [32]. According to our literature review, this study is considered as an outlying study; however, it was not excluded and included in our analysis.

Dai et al. studied the chest CT and clinical features of 234 SARS-CoV-2 laboratory-confirmed cases. Six (6/234, 2.5%) patients had initially negative RT-PCR and 15/234 (6.4%) patients were without abnormal lung changes by CT. Thus, the reported chest CT sensitivity is 93.6% [33]. Likewise, Ding et al. studied the chest CT findings by duration of symptoms, 17/112 (15%) laboratory-confirmed cases with different stages of symptomatic COVID-19 infection showed no lung abnormalities. Thus, the reported sensitivity for chest CT is (84.8%) [34]. Additionally, Hu et al. studied the chest CT features for 46 laboratory-confirmed COVID-19 cases. Two cases were negative according to the first chest CT and were positive on the second/follow-up CT, with reported sensitivity 95.6% [35].

### 4.2. Analysis of Case Series

Bhat et al. reported case series of 8 confirmed cases from USA. They used chest radiography as the initial diagnostic tool and Chest CT was not performed for all cases. Case no. 1 showed initially negative swab for COVID-19 with positive CT findings, the RT-PCR testing turned positive when repeated (2nd time). Case no. 2 showed initially negative RT-PCR with positive findings by chest radiography (chest CT not performed); the nucleic acid testing turned positive on 8th day. Interestingly, the authors pointed to the American College of Radiology recommendations to use chest imaging in specific clinical conditions and not to screen or diagnose COVID-19 infections. Additionally, a significant overlap in imaging findings with other viral, bacterial, or organizing pneumonias; collagen vascular disease; and drug toxicities should be considered [36].

Lescure et al. reported a case series for 5 confirmed cases from France. Two patients (case nos. 4 and 5) showed normal chest imaging (by CXR, CT not performed) despite positive RT-PCR and high viral load [37].

### 4.3. Analysis of Case Reports

Xu et al. described the chest CT features of three female patients with COVID-19 pneumonia and initial negative results for RT-PCR. Patient no.1 in their study showed progressive changes in chest CT (bilateral multifocal fusion structure of GGOs and multinodular infiltration) despite negative RT-PCR. The chest CT of patient nos. 2 and 3 both showed mild pneumonia. The RT-PCR was still negative for the three patients till the 6^th^ to 8^th^ day after the onset of the symptoms. The authors stated that the RT-PCR testing of the nasopharyngeal swab probably is not sensitive for COVID-19 at early stages of clinical presentation and thus chest CT may be helpful for early detection of severe or critical cases [38].

Li et al. reported two out of ten initially negative patients were confirmed to be positive for COVID-19 infection offering about 20% false-negative rates for RT-PCR testing. They declared that from a clinical point of view, chest CT scan could be utilized as the first and immediate tool for the physicians to screen the highly suspected cases and to take a necessary action while RT-PCR serves as a confirmatory tool [39].

Hao and Li reported a case of 56-year-old male presented with hyperthermia 39.1°C. Chest CT scan revealed bilateral multiple GGOs. However, three of RT-PCR assays of oropharyngeal swabs for SARS-CoV-2 were negative. Repeated chest CT showed significant progression of multifocal GGOs and mixed consolidation at peripheral area of both lungs. They repeated the RT-PCR testing for the fourth time and it was positive. The patient was finally diagnosed with COVID-19 infection. The authors mentioned that they performed all RT-PCR experiments in strict accordance with the standard protocols. They explained this finding by suggesting a considerable increase in the virus load, which is related to the deterioration of the patient's condition. The authors declared that RT-PCR testing cannot withstand the needs of the rising number of infected population due to its relatively long processing time and the presence of insufficient viral particles in the specimens [40].

Huang et al. reported a case of 36-year-old man with clinical features coincides with COVID-19 infection. Chest CT scan showed bilateral multiple peripheral GGOs. However, the initial RT-PCR test of the sputum sample was negative for the SARS-CoV-2. Repeated chest CT three days following admission showed progression of GGOs to more consolidation. RT-PCR testing was repeated and the result was negative for the second time. Finally, the third RT-PCR test turned positive on the 6th day after admission. The authors recommended that the probability of a false-negative RT-PCR result should be taken into account in the context of patient's recent history of exposure and the presence of clinical signs and symptoms coincide with COVID-19 infection [41].

Park et al. reported a case of 10-year-old Korean girl with initially negative RT-PCR for three times but with abnormal chest CT findings despite mild symptoms [42]. Duo et al. also reported a 56-year-old male with symptoms coincides with COVID-19 but initially negative two RT-PCR tests and chest CT with multifocal GGOs [43]. Furthermore, Burhan et al. reported a 47-year-old man with initially negative RT-PCR and positive chest CT findings [44].

### 4.4. Considerations regarding the Initially Negative RT-PCR Testing

The initially negative RT-PCR test for SARS-CoV-2 infection may be explained through inappropriate maneuver of sample collection, viral dynamics, or certain PCR techniques. For instance, the recommended steps for collection of good quality nasopharyngeal samples include insertion of the swab through the nostril parallel to the palate, keeping the swab in its position for several seconds followed by instant placement of the swab into a sterile tube containing a 2 to 3 mL of viral transport medium [45].

On the other hand, for collection of oropharyngeal specimens, insertion of the swab into the posterior pharynx, avoid touching the tongue, and then instant insertion of the swab into a sterile tube, also containing a 2 to 3 mL of viral transport medium are the suggested measures to obtain proper oropharyngeal (e.g., throat) specimens. A major cause of SARS-CoV-2 RT-PCR diagnostic errors may be related to the inability to fulfill the recommended sampling measures as well known for other viral diseases [46].

With regards to viral dynamics, two gray zones could be recognized and might result in false-negative RT-PCR tests. The first one is attributed to the low viral load in patients with mild or no symptoms. The second one would rather represent the SARS-CoV-2 tail of infection, when there is symptoms alleviation. In this final stage of infection, shedding of the virus may continue, though remaining below the lower detection limit of some RT-PCR tests [47].

A further indispensable issue is the likelihood of active mutations owing to the fact that RNA-dependent RNA polymerases of corona viruses are error-prone. Such viral evolvement may also diminish the accuracy of RT-PCR tests [48, 49]. The reliability of RT-PCR testing can also be affected by absent or insufficient harmony of primers and probes as well as by a variety of technical and analytical errors [50, 51]. For example, Pan et al. discussed the potential that thermal inactivation of SARS-CoV-2 at 56°C could theoretically disturb the viral nucleic acid integrity and cause false negatives in RT-PCR tests [52].

### 4.5. Considerations regarding Chest CT

Chest CT is one of the most widespread imaging techniques in clinical use worldwide. However, CT has numerous known side effects.

#### 4.5.1. Radiation Dosage

Repeated chest CT usage can expose the patient to unnecessary doses of radiation. Currently, there is no certain recommended radiation dose level for chest CT in patients with COVID-19 pneumonia. However, using less kilo-voltage settings will result in lower radiation doses together with approximately equal diagnostic quality compared to full radiation dose CT [53, 54].

#### 4.5.2. Contrast-Induced Nephropathy

Contrast-induced nephropathy, a leading cause of hospital-acquired acute renal injury, is a major concern associated with contrast-enhanced CT. Most studies therefore support the usage of single phase, low radiation dose, non-contrast chest CT. If there is a clinical deterioration of cardiorespiratory status or suspected pulmonary embolism in patients with COVID-19 pneumonia, then a postcontrast chest CT could be beneficial [55].

#### 4.5.3. Fast Scanning Techniques

For patients suffering from dyspnea or coughing and consequently cannot hold their breath during image acquisition in order to diminish motion artifact, faster scanning protocols should be considered by reducing the rotation time of the tube detector system in conjunction with faster gantry rotation time (0.5 s or less) and higher pitch values (more than 1 : 1). However, the potential to apply faster scanning relies on the model of the CT scanner together with the patient's body habitus [56].

In addition, it is essential to notice that the excessive demand for chest CT scan will increase its cost and carry a significant risk of transmission and contamination. Magidi and Niksolat mentioned several considerations for emergency and radiology departments during work up for patients with COVID-19. Among these considerations are the safety of HCWs and decontamination of the radiology equipment. They recommended a time line of 30 minutes to 1 hour to allow decontamination and cleaning of the equipment using approved methods. Moreover, the CT scan should be performed at areas with less traffic to avoid risk of transmission to uninfected patients and medical staff [57].

### 4.6. Chest CT vs. RT-PCR Test

After reading and analyzing all what have been published on this topic, we add our voice to the recommendations published by the Radiology Scientific Expert Panel that COVID-19 RT-PCR test has a high specificity but lower sensitivity which is reported to be as low as 60% to 70%. Thus, multiple negative RT-PCR tests are required to exclude the diagnosis of COVID-19 [58].

Based on many observations of lung abnormalities on chest CT before conversion to positive RT-PCR, the Chinese health authorities initially extended the official case definition to include patients with typical findings at chest CT despite the initially negative RT-PCR test which has resulted in a higher number of COVID-19 suspected cases. However, the presence of no or mild chest CT findings in early stages of infection spotlights the challenges of early diagnosis [58].

Finally, we would like to point to the study by Liu and Li which demonstrated that the typical GGOs detected by chest CT and seen in almost COVID-19 patients are not due to direct viral attacks to the lungs. Liu and Li explained that the virus attacks the 1-beta chain of hemoglobin to dissociate the heme resulting to less and less hemoglobin that carry oxygen and exchange carbon dioxide. Thus, the lungs will have extensive inflammation resulting in ground glass such as lung image [59].

### 4.7. Chest Radiography

The chest CT offers greater sensitivity for detection of early pneumonic changes in comparison to CXR which estimated to be normal in up to 63% of patients with COVID-19 pneumonia, particularly in their early stages of the infection [60]. In agreement, The Fleischer society for thoracic radiology released a multinational consensus statement which advocated that the usage of CXR is not beneficial in mild or early COVID-19 infection; however, it could be helpful for monitoring the rapid progression of lung abnormalities in COVID-19 critical cases admitted to intensive care units [61]. Additionally, it is advised that, whenever possible, a posterior-anterior CXR view should be requested as it provides a better image than an anterior-posterior view [60].

### 4.8. Hazards and Toxicities of Chest Imaging

Chest imaging for detection of COVID-19 pneumonia, either with CXR or CT, is associated with radiation exposure of the patient. The hazards of repeated exposure to ionizing radiation have to be considered. Many studies have shown no difference in important outcomes (e.g., morbidity, mortality, and length of ICU stay) for patients imaged on demand as compared with daily routine protocols [62, 63].

Some reactions occur when biological tissues are exposed to ionizing radiation used in both CXR and CT. The uniquely high energetic ionizing radiation is capable to overcome the electrons binding energy, resulting in production of hydroxyl radicals. The emission of these hydroxyl radicals will result in interaction with nearby DNA causing DNA double-strand breaks and/or base damage. Furthermore, they can directly ionize DNA. On the other hand, the cell quickly fixes most radiation-induced damages; however, DNA double-strand breaks are less rapidly repaired, and misrepair can occasionally occur leading to point mutations, chromosomal translocations, and gene fusions, all of which are related to cancer induction [64].

Levels of radiation exposure from CXR may range from 0.06 to 0.25 mSv according to the number of views taken, the signal to noise ratio in the digital system, the voltage, and the film-screen system. As compared with plain film radiography, CT necessitates much higher doses of radiation, resulting in a marked increase in radiation exposure. The average radiation dose for chest CT varies from 3 to 27 mSv [65].

According to the international commission of radiological protection, in a publication from 1990, there is a 5% increased risk of malignancy induction for 1 Sv radiation exposure level. Thus, application of low dose radiation chest CT is essential by reduction of the tube current to 10–50 mAs instead of the standard 80 and 300 mAs, increasing the table increment thus reducing the exposure time and subsequently the exposure levels, together with the reduction of the tube voltage [65, 66].

### 4.9. Limitations

This meta-analysis has some limitations: (1) the analysis included multiple retrospective studies; (2) most studies are from China and it would be better to include other studies with a broad geographic scope when available in the future; (3) there was a lack of some data and some were calculated indirectly from percentages and other provided parameters; (4) the wide range of chest CT sensitivity is due to the presence of one outlying study which results in conflicts with the rest of the studies.

## 5. Conclusion

Chest CT examination is a sensitive method for SARS-CoV-2 identification, while RT-PCR testing may yield false-negative results. Therefore, we advocate that patients with positive image findings but negative RT-PCR should be isolated and RT-PCR should be repeated to avoid misdiagnosis. Additionally, a normal chest CT scan cannot exclude the diagnosis of COVID- 19. Standardized infection control and prevention practices for all patients with respiratory illness during chest CT scan should be carried out.

## Figures and Tables

**Table 1:** Characteristics of the included studies.

Author	Journal	Date (month/year)	Country	Study type	*N*	Age	Sex	Quality score	Reference
Ai et al.	Radiology	Feb/2020	China	Retrospective cohort	1014	51 ± 15	M: 467 (46%) F: 547 (54%)	10	[6]
Fang et al.	Radiology	Feb/2020	China	Retrospective cohort	51	Median: 45 IQR 39–55	M: 29 (56.9%) F: 22 (43.1%)	10	[20]
Xi et al.	Radiology	Feb/2020	China	Retrospective cohort	167	NR	NR	10	[21]
Bernheim et al.	Radiology	Feb/2020	China	Retrospective cohort	121	45.3 ± 16	M: 61 (50%) F: 60 (50%)	12	[28]
Wu et al.	Clinical Infectious Diseases	Feb/2020	China	Retrospective cohort	80	Median: 46.1	M: 39 (48.7%) F: 41 (51.3%)	13	[29]
Chung et al.	Radiology	Feb/2020	China	Retrospective cohort	21	51 ± 14	M: 13 (62%) F: 8 (38%)	11	[11]
Shi et al.	The Lancet Infectious Diseases	Feb/2020	China	Retrospective cohort	81	49.5 ± 11	M: 42 (52%) F: 39 (48%)	11	[14]
Yang et al.	Journal of Infection	Feb/2020	China	Retrospective cohort	149	45.1 ± 13.3	M: 81 (54.4%) F: 68 (45.6%)	12	[24]
Guan et al.	New England Journal of Medicine	Feb/2020	China	Retrospective cohort	1099	Median: 47 IQR 35-58	M: 639 (58.1%) F: 460 (41.9%)	13	[31]
Cheng et al.	American Journal of Roentgenology	Feb/2020	China	Retrospective cohort	33 11+ve COVID-19 22 Non -COVID	50.36 ± 15.5 for cases	M: 8 (72.7%) F: 3 (27.3%)	10	[12]
Huang et al.	The Lancet Infectious diseases	Feb/2020	China	Prospective cohort	41	Median: 40 IQR 41-58	M: 30 (73.2%) F: 11 (26.8%)	12	[13]
Long et al.	European Journal of Radiology	March/2020	China	Case control study	87 Cases: 36 Control: 51	44.8 ± 18.2 for cases	Cases: 36 M: 20 (55.6%) F: 16 (44.4%)	10	[22]
Himoto et al.	Japanese Journal of Radiology	March/2020	Japan	Retrospective cohort	21 (cases: 6, others: 15)	Median: 58.5 Range (45–81) for 6 cases	M: 5 (83.3%) F: 1 (16.7%)	10	[18]
Zhang et al.	European Respiratory Journal	March/2020	China	Retrospective cohort	17	Median: 48.6 Range (23–74)	M: 8 (47%) F: 9 (53%)	11	[19]
Xu et al.	International Journal of Infectious Diseases	March/2020	China	Retrospective cohort	51	NR	M: 25 (49%) F: 26 (51%)	10	[26]
Guan et al.	Academic Radiology	March/2020	China	Retrospective cohort	53	42	M: 25 (47.2%) F: 28 (52.8%)	10	[27]
Caruso et al.	Radiology	April/2020	Italy	Prospective cohort	158	57 ± 17	M: 83 (52.5%) F: 75 (47.5%)	12	[25]
Wang et al.	International Journal of Infectious Diseases	April/2020	China	Retrospective cohort	125	41.46 ± 15	M: 71 (56.8%) F: 54 (43.2%)	10	[30]
Meng et al.	Journal of Infection	April/2020	China	Retrospective cohort	58	42.6 ± 16.5	M: 26 (44.8%) F: 32 (55.2%)	11	[17]
Zhifeng et al.	Journal of Clinical Virology	April/2020	China	Retrospective cohort	69	Range (23–82)	M: 41 (59.4%) F: 28 (40.6%)	9	[32]
Xia et al.	Journal of Clinical Virology	April/2020	China	Retrospective cohort	10	56.5 ± 11.16	M: 4 (40%) F:6 (60%)	10	[15]
Dai et al.	International Journal of Infectious Diseases	April/2020	China	Retrospective cohort	234	44.6 ± 14.8	M: 136 (58.1%) F: 98 (41.9%)	12	[33]
Ding et al.	European Journal of Radiology	April/2020	China	Retrospective cohort	112	55.8 ± 16.1	M: 51 (45.5%) F: 61 (54.5%)	11	[34]
Hu et al.	European Journal of Radiology	April /2020	China	Retrospective cohort	46	39.2 ± 9.6 Range (23–60)	M: 27 (58.7%) F: 19 (41.3%)	10	[35]
Wang et al.	Clinical Radiology	May/2020	China	Retrospective cohort	114	Median: 53 Range (23–78)	M: 56 (49.1%) F: 58 (50.9%)	11	[23]
Li et al.	Journal of Clinical Virology	June/2020	China	Retrospective cohort	225	50 ± 14	M: 120 (53.3%) F: 105 (46.7%)	10	[16]

N = number of cases; NR = not reported; M = male; F = female.

**Table 2:** Characteristics of the included case reports and case series.

Author	Journal	Date (month/year)	Country	Study design	*N*	Age	Sex	Reference
Burhan et al.	Indonesian Journal of Internal Medicine	Jan/2020	Indonesia	Case report	1	47 y male	M:1 (100%)	[44]
Li et al.	Korean Journal of Radiology	Feb/2020	China	Case report	2	36 y male, 10 mon. boy	M:2 (100%)	[39]
Haung et al.	Radiology	Feb/2020	China	Case report	1	36 y	M:1 (100%)	[41]
Hao et al.	Travel Medicine and Infectious Diseases	March/2020	China	Case report	1	56 y	M:1(100%)	[40]
Xu et al.	Clinical Infectious Diseases	March/2020	China	Case report	3	52.3 ± 11 Median: 47	F: 3 (100%)	[38]
Park et al.	Journal of Korean Medical Science	March/2020	Korea	Case report	1	10 years	F: 1 (100%)	[42]
Dou et al.	European Journal of Radiology	March/2020	China	Case report	2	56 y male 21 y female	M: 1 (50%) F: 1 (50%)	[43]
Bhat et al.	Current Problems in Diagnostic Radiology	April/2020	USA	Case series	8	54.5 ± 11.5	M: 6 (80%) F: 2 (20%)	[36]
Lescure et al.	Lancet Infectious Diseases	March/2020	France	Case series	5	Median: 46 Range: 30–80	M: 3 (60%) F: 2 (40%)	[37]

mon = month; N = number of cases; y = years; M = male; F = female.

**Table 3:** Performance of chest CT for COVID-19 infection compared to RT-PCR.

Author	TP	TN	FP	FN	Sensitivity (95% CI)	Specificity (95% CI)	PPV (95% CI)	NPV (95% CI)	Reference
Ai et al.	580	105	308	21	97% (95–98) RT-PCR 70%	25% (22–30)	65% (62–68)	83% (76–89)	[6]
Fang et al.	50/51	0	0	1/51	98% (90–100)	—	—	—	[20]
Xie et al.	155/167	0	5/167	7/167	95.68% (91–98)	—	—	—	[21]
Long et al.	35/36	0	0	1/36	97.2% (85.4–99.9) RT-PCR 83.3%	—	—	—	[22]
Chung et al.	18/21	0	0	3/21	85.7% (64 to 97)	—	—	—	[11]
Bernheim et al.	94/121	0	0	27/121	77.6% (69 to 85)	—	—	—	[28]
Himoto et al.	6/6	0	15/21	0	100% (54–100)	—	—	—	[18]
Zhang et al.	17/17	0	0	0	100% (80–100)	—	—	—	[19]
Caruso et al.	60	54	42	2	97% (89–99)	56% (45–66)	59% (53–64)	96% (87–99)	[25]
Wang et al.	120/125	0	0	5/125	96% (91–98)	—	—	—	[30]
Wang et al.	110/114	0	0	4/114	96.4% (91–99)	—	—	—	[23]
Shi et al.	81/81	0	0	0	100% (95–100)	—	—	—	[14]
Yang et al.	132/149	0	0	17/149	88.5% (82–93)	—	—	—	[24]
Guan et al.	869/1099	0	0	230/1099	79% (76–81)	—	—	—	[31]
Guan et al.	47/53	0	0	6/53	88.6% (77–96)	—	—	—	[27]
Meng et al.	58/58	0	0	0	100% (94–100)	—	—	—	[17]
Cheng et al.	11/11	0	0	0	100% (71–100)	—	—	—	[12]
Huang et al.	41/41	0	0	0	100% (91–100)	—	—	—	[13]

Zhifeng et al.	NR	NR	NR	NR	12% (4.6–24.3) For PCR 30.16% (19.2–43)	100% (82–100) For PCR 100% (54.1–100)	—	—	[32]

Li et al.	225/225	0	0	0	100% (98–100)	—	—	—	[16]
Wu et al.	55/80	0	0	25/80	68.7% (57–78)	—	—	—	[29]
Xu et al.	39/51	0	0	12/51	76.5% (62–87)	—	—	—	[26]
Xia et al.	10/10	0	0	0	100% (69–100)	—	—	—	[15]
Dai et al.	219/234	0	0	15/234	93.6% (89.6–96.3)	—	—	—	[33]
Ding et al.	95/112	0	0	17/112	85% (76.81–90.9)	—	—	—	[34]
Hu et al.	44/46	0	0	2/46	95.6% (85–99.4)	—	—	—	[35]

TP = true positive; TN = true negative; FP = false positive; FN = false negative; PPV = positive predictive value; NPV = negative predictive value, NR = not reported.

## Data Availability

This is a meta-analysis; all the included studies are mentioned in the references.

## Abbreviations

COVID-19: Corona virus disease-2019
CSG: Coronavirus study group
CT: Computed tomography
CXR: Chest X-ray
GGOs: Ground glass opacities
HCWs: Health care workers
IHE: Institute of Health Economics
PHEIC: Public Health Emergency of International Concern
RT-PCR: Real time reverse transcription polymerase chain reaction
SARS-CoV-2: Severe acute respiratory syndrome coronavirus-2
WHO: World Health Organization.
